# Prediction of the Ecological Behavior of *Burkholderia gladiolus* in Fresh Wet Rice Noodles at Different Temperatures and Its Correlation with Quality Changes

**DOI:** 10.3390/foods14081291

**Published:** 2025-04-08

**Authors:** Mengmeng Li, Ke Xiong, Wen Jin, Yumeng Hu

**Affiliations:** 1 Beijing Engineering and Technology Research Centre of Food Additives, Beijing Technology & Business University (BTBU), Beijing 100048, China2130022076@st.btbu.edu.cn (W.J.); 2130022072@st.btbu.edu.cn (Y.H.); 2 Beijing Laboratory for Food Quality and Safety, Beijing Technology & Business University (BTBU), Beijing 100048, China; 3 Beijing Innovation Centre of Food Nutrition and Human, Beijing Technology & Business University (BTBU), Beijing 100048, China

**Keywords:** Bongkrekic Acid, food safety, growth dynamics, probabilistic model, rice and flour products

## Abstract

*Burkholderia gladioli pathovar cocovenenans* (BGC) is a highly lethal foodborne pathogen responsible for outbreaks of food poisoning with the highest recorded mortality rates among bacterial foodborne illnesses in China. In this study, the ecological behavior of BGC and its Bongkrekic Acid (BA) production dynamics in fresh wet rice noodles (FWRN) were investigated under isothermal conditions ranging from 4 °C to 37 °C. Growth kinetics were modeled using the Huang, Baranyi, and modified Gompertz primary models, with secondary models (Huang square root model and Ratkowsky square root model) describing the influence of temperature on growth parameters. Among these, the Huang–Huang model combination exhibited the best performance, with a root mean square error (RMSE) of 0.009 and bias factor (*B_f_*) and accuracy factor (*A_f_*) values close to 1. Additionally, we examined the impact of BGC contamination on the quality attributes of FWRN, including pH, color (L*, a*, b*), hardness, and moisture content. The results indicated that BGC growth significantly increased pH and yellowing (b*) values, while changes in texture and moisture were less pronounced. A probabilistic model was further developed to predict BA production under various temperature scenarios, revealing that BA formation was most likely to occur between 24 °C and 30 °C. While this study provides valuable predictive tools for microbial risk assessment and quality control of FWRN, limitations include the exclusion of additional environmental factors such as oxygen and relative humidity, as well as the lack of direct investigation into the degradation behavior of BA. Future research will expand model parameters and include sensory evaluations and advanced microbiological analyses to enhance applicability under real-world storage and transportation conditions.

## 1. Introduction

Fresh wet rice noodles (FWRN) are a popular cereal product in most regions of Southeast Asia, China, and Japan [1,2]. Due to their high-water activity and nutritional composition, FWRN, which originate from various countries, carry a potential risk of microbial contamination, such as *Bacillus cereus* [3,4]. In the 1977 yeast rice noodle poisoning incident, Chinese researchers identified a novel pathogenic bacterium known as *Pseudomonas cocovenenans* subsp. *farinofermentans. Pseudomonas coconuts*, a subspecies, is present in rice noodles for the first time, presenting a notable and significant risk. After comparing the 16S rDNA sequences of *Pseudomonas cocovenenans* subsp. *Farinofermentans*, along with *Burkholderia cocovenenans* and *Burkholderia gladioli*, it has been classified as the fourth pathogenic type of *Burkholderia gladiolus* [5,6]. It was named *Burkholderia gladioli pathovar cocovenenans* (BGC).

BGC is a Gram-negative, aerobic bacterium that produces the mitochondrial toxin Bongkrekic Acid (BA), which is a tricarboxylic fatty acid. The oral LD50 of BA in mice is 3.16 mg/kg [7]. Human consumption of it can lead to vomiting, diarrhea, abdominal pain, and other symptoms. To date, it has been identified as the food-borne pathogen with the highest fatality rate in China, up to 40–100% [8]. Nonetheless, the degradation pathway of BA remains unknown. BGC has now been associated with numerous outbreaks of foodborne illness. Contaminated foods mainly consist of rice and noodle products that have had prolonged fermentation or spoilage (such as sour dumplings, glutinous rice dumplings, and wet noodles), coconut products, and prolonged soaking of fungus and ginkgo ears [9,10]. Incidents have primarily occurred in Indonesia and certain regions of China, resulting in nearly 3000 cases of BA poisoning (related to fermented coconut milk products) in Indonesia, with a mortality rate of approximately 60% [11,12,13]. Based on the available records, from 1953 to 1994, a total of 545 incidents of BGC food poisoning occurred in 16 provinces across China, resulting in 3352 cases of poisoning and 1401 fatalities. However, from 2005 to 2020, there were 30 reported cases of poisoning, with 188 cases resulting in poisoning and 85 fatalities [14]. From 2018 to 2020, Guangdong Province reported five cases of food-related BA poisonings involving river noodles, resulting in the poisoning of 21 people and nine fatalities, with a case-fatality rate of 42.9% [15].

Bongkrekic Acid (BA), produced by Burkholderia gladioli pathovar cocovenenans (BGC), is a highly potent mitochondrial toxin that inhibits the adenine nucleotide translocator (ANT), disrupting ATP synthesis and leading to irreversible cell damage. Reported lethal doses in humans are as low as 1–1.5 mg, and outbreaks of BA-related food poisoning have been associated with mortality rates exceeding 40% in affected populations. Documented incidents in China and Indonesia have involved traditional fermented products and starch-rich foods such as corn flour cakes and rice noodles, often under warm and humid conditions that favor BGC proliferation and toxin synthesis. For instance, a well-documented outbreak in Yunnan Province (China) in 2020 resulted in 9 deaths out of 12 individuals after ingestion of a fermented rice-based dish contaminated with BA.

FWRN is a rice product that has undergone a series of production processes, including washing, soaking, milling, ripening, molding, cooling, packaging, and others [16,17], that is susceptible to contamination by BGC in the raw material as well as in the processing and distribution process. The study showed that in 129 samples of rice, broken rice, and starch from southern provinces, six strains of *Burkholderia gladioli* were isolated from imported broken rice, of which four strains produced BA [18]. However, when 1570 samples of rice noodle and starch products from Guangdong Province were tested, it was discovered that five samples contained *Burkholderia gladioli*, with one of them producing BA [19]. Furthermore, Zhu et al. [20] collected a grand total of 276 samples from the production process (including raw materials, environmental samples, and finished products) and distribution of rice and noodle products. Their findings revealed that no contamination of *Burkholderia* spp. was identified in either the milling or cooking plants. However, they identified 25 strains of *Burkholderia gladioli* in 180 food samples that originated mainly from rice during the production and distribution process. This suggests that the BGC contamination in FWRN primarily originated from the raw materials and end products used in production. It is noteworthy that source contamination can be easily transmitted to all subsequent stages. Furthermore, the processing of FWRN typically does not involve sterilization and has only a short shelf life of a few days [21]. Additionally, its quality can be easily affected by transportation and storage conditions, such as temperature and time. The growth of microorganisms in contaminated food is closely linked to its quality. Microorganisms have the ability to multiply within food and impact several quality factors, including its pH, moisture content, color, and texture.

At present, the primary emphasis of BGC research is directed towards the identification of the metabolite BA, with limited attention devoted to conducting comprehensive studies on the growth and toxicity-generating attributes of BGC. Assessing the risk posed by environmental changes to food microorganisms using traditional bacterial quantification methods requires substantial time, money, and resources. On the other hand, predictive microbiology is a mathematical tool that assists in determining the likelihood of microbial growth. Without the need for microbiological testing, it can predict the growth and death of microorganisms, thereby offering a crucial safeguard for ensuring food safety. While there have been numerous studies on predictive modeling of microorganisms in meat and vegetable products, both domestically and internationally [22,23,24], there has been a relative lack of research on rice. Juneja et al. [3] and Hwang and Huang [25] investigated the growth and survival of *Bacillus cereus* in the pasta cooling process and cooked rice using predictive modeling, respectively. However, there is a gap in the research on modeling of BGC growth and toxin production in rice and noodle foods such as FWRN. Thus, the aim of this study was to examine the development of BGC in FWRN under constant temperature conditions by employing predictive microbiology models. Additionally, we aimed to uncover the direct correlation between the level of BGC and the yield of BA by constructing both kinetic and probabilistic models and subsequently validating the predictive model. Furthermore, we analyzed the correlation between the growth of microorganisms and the pH, color, texture, and moisture content of the system, while also assessing the quality of FWRN. The relationship between microbial growth and factors such as pH, color, texture, and moisture content were analyzed.

In this study, temperature was selected as the key environmental factor influencing the growth and toxin production of BGC in fresh wet rice noodles, primarily because temperature is the most critical and variable factor during the processing, transportation, and storage of FWRN. Although other environmental factors such as relative humidity and oxygen concentration can also significantly affect the behavior of BGC and the biosynthesis of BA, they were not the focus of this study. This is because FWRN are a semi-closed system, where oxygen availability is relatively stable during short-term storage, and relative humidity is generally high due to the product’s intrinsic high-water content and packaging characteristics. However, we acknowledge that microbial ecology is often influenced by the interaction of multiple environmental parameters. Future studies will incorporate a broader range of factors, including relative humidity, oxygen concentration, and packaging atmosphere (e.g., vacuum, modified atmosphere packaging), to develop more comprehensive and robust predictive models. This would help to better simulate real-world storage and distribution conditions and further enhance the accuracy and applicability of risk assessments for BGC contamination in FWRN.

It is worth noting that the degradation pathway of BA remains largely unexplored, which limits our understanding of its persistence and fate in food matrices such as FWRN. The absence of detailed studies on BA degradation may affect the practical applicability of this study, as it restricts our ability to fully assess the dynamics of toxin accumulation and potential detoxification during food processing and storage. While this study focused on predicting the growth behavior of BGC and the production kinetics of BA, the inability to account for BA degradation might lead to conservative risk assessments, assuming that all produced toxins persist throughout the product’s shelf life. Future research should explore the physicochemical and biological conditions (e.g., pH variations, enzymatic activity, thermal processing) that may contribute to the degradation or inactivation of BA. Integrating such data would provide a more holistic risk assessment framework and contribute to the development of effective strategies for mitigating BA-related food safety risks in rice- and flour-based products.

This study is of high importance given the frequent detection of BGC in rice-based food products and the severe consequences of BA contamination. The parameters analyzed—including microbial growth kinetics, toxin formation, and their interaction with food quality attributes such as pH, color, and texture—are crucial for developing predictive tools that support food safety risk assessment and shelf-life management. Understanding how environmental factors influence both microbial behavior and product quality allows stakeholders to design targeted interventions (e.g., temperature control, packaging strategies) and avoid conditions that favor BA production. By focusing on fresh wet rice noodles (FWRN), a widely consumed and highly perishable product in Asia, this research provides practical insights for food manufacturers, regulators, and public health authorities.

## 2. Materials and Methods

### 2.1. Strain and Inoculum Preparation

The freeze-dried powder of BGC ATCC33664 (Taisituo Biological Corporation, Ningbo, China) stored in the refrigerator at 4 °C was activated. The medium was then streaked onto modified potato dextrose agar (mPDA) (Hope, Qingdao, China) and temporarily stored in a 4 °C refrigerator. Before use, a ring of mPDA was selected and inoculated into sterile test tubes containing 10 mL tryptose soya broth (Aoboxing, Beijng, China) and cultured in a shaking bed at 37 °C and 180 r/min for 18 h to the later stage. After coating on the mPDA plate, the concentration of the original bacterial solution is 1 × 10^9^ CFU/mL. Then, sterile normal saline (0.85% NaCI solution) was used for 10-fold series dilution of the bacterial suspension, and 1 × 10^8^ CFU/mL bacterial solution was selected for subsequent inoculation.

### 2.2. Sample Preparation and Inoculation

The FWRN samples were purchased from supermarkets (Guangxi, China) and had been pasteurized during production. As an additional safety measure, FWRN were microwaved for 1 min prior to the experiment for secondary sterilization. FWRN were cut into long strips of about 2 cm and then dispensed into sterile homogenized bags (Bkman, Changde, China) of 10 g FWRN each.

Add 1 mL bacterial suspension to FWRN by dot connection and gently knead the homogenizing bag to mix FWRN with the bacterial suspensions well, leaving a small opening for the BGC to grow. An initial concentration of 1 × 10^7^ CFU/g was obtained. The purpose of using higher inoculum doses was to maintain the dominance of BGC in the FWRN and to gain a more complete understanding of the ecological behavior of BGC in the FWRN. Each growth test was repeated three times independently, and 1 mL of sterile saline was added to 10 g FWRN as a blank control group.

### 2.3. Storage Conditions and Bacterial Counts

The inoculated samples were placed in a constant temperature incubator for incubation. The different temperature settings were 4 °C, 16 °C, 20 °C, 24 °C, 30 °C, and 37 °C. The samples were homogenized in a tapping homogenizer for 2 min after adding 90 mL of sterile saline, tapping 8 times per second. A tenfold series dilution was made in saline, and 0.1 mL was taken and spread evenly on mPDA. The plate was incubated in 37 °C incubator for 48 h. The count was recorded as lg CFU/g and converted to log N(t).

The selection of intermediate temperatures (16 °C, 20 °C, and 24 °C) in this study was based on commonly encountered conditions during the distribution and short-term storage of FWRN in regions of Southeast Asia and southern China. According to surveys on the rice noodle supply chain, these temperatures reflect ambient conditions in local retail markets, transportation vehicles without refrigeration, and storage environments during the warmer months, where air-conditioning or cold chain measures may not always be employed. Furthermore, temperatures within this range are frequently observed during household storage when refrigeration is not used. Nevertheless, we acknowledge that including additional temperatures, such as 10 °C—representing sub-optimal refrigeration—and 35 °C—representing extreme ambient conditions during summer or inadequate temperature control during transportation—would strengthen the model’s relevance to a broader spectrum of real-world scenarios. In future studies, we intend to expand the experimental design to cover these additional conditions to further enhance the generalizability and practical application of the predictive models developed herein.

### 2.4. Quality Change in Fresh Wet Rice Noodles

#### 2.4.1. Texture Determination

The hardness determination of FWRN was modified from the study of Tantala et al. [26] and Geng et al. [27]. Three FWRN samples of 5 cm length and similar diameter (about 1.8 mm) were taken and placed on the test bench of the texture analyzer, and the hardness test was carried out at equal intervals using a cylindrical probe (35 mm diameter). The specific parameters were as follows: 1.0 mm/s test speed, 50% compression ratio, 0.04905 N trigger force. Each sample was measured ten times and averaged.

#### 2.4.2. Color

A colorimeter (Konica Minolta, CR-400, Tokyo, Japan) was used to measure the color of FWRN and record L* (brightness), a* (red), and B* (yellow) values.

#### 2.4.3. PH

Ninety milliliters of distilled water was added to the FWRN sample (10 g), which was then mashed and homogenized until uniform [28]. The pH of the supernatant was measured using a pH meter (Leici, PHSJ-5, Shanghai, China).

#### 2.4.4. Moisture Content

We weighed 10 g FWRN, spread it flat on the sample tray of the rapid moisture meter (Mettler Toledo, HE53, Shanghai, China), and heated it at 105 °C to determine the moisture content of FWRN.

### 2.5. Bongkrekic Acid Analysis

**Figure 1:**
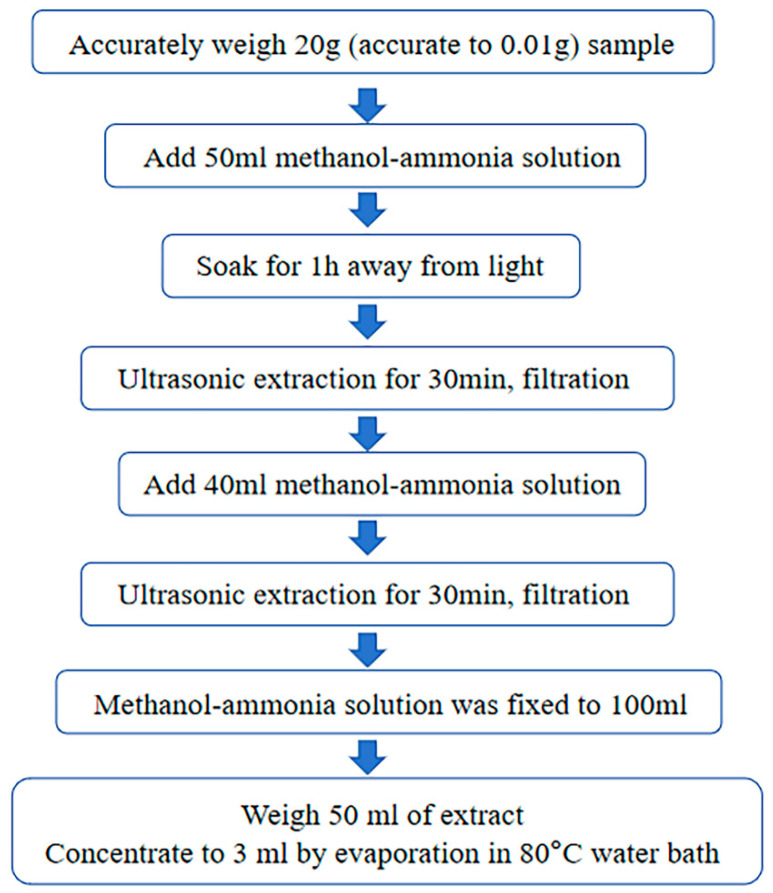
BA extraction process in FWRN.

FWRN inoculated with BGC were incubated in a thermostatic incubator at 4 °C, 16 °C, 20 °C, 24 °C, and 30 °C, and the accumulation of BA in FWRN at different temperatures was determined using liquid chromatography at 2 h, 4 h, 6 h, 8 h, 10 h, 12 h, 1 d, 3 d, 5 d, 7 d, and 9 d (*n* = 3). The quantitative detection of BA was carried out according to the “Determination of Bongkrekic acid in foods” published by the National Standardization Administration of China in 2023, with a detection limit of 5 μg/kg [29]. The extraction process of BA in FWRN is shown in Figure 1. High performance liquid chromatograph (HPLC) (Waters, Milford, MA, USA); Ultrasonic oscillator (Shumei, KQ5200DA, Cangzhou, China).

### 2.6. Model Development

#### 2.6.1. Primary Models

We fit three commonly used growth models to BGC growth data in FWRN at different temperatures: the Baranyi, Huang, and modified Gompertz models [30,31,32]. The nonlinear least square method in Python 3.10 is used to fit the function and find the optimal curve.

(1) $$y\left(t\right)=y_{0}+\mu_{max}A\left(t\right)-ln\left[1+\frac{e^{\mu_{max}A\left(t\right)}-1}{e^{\left(y_{max}{-y}_{0}\right)}}\right]$$

(2) $$A\left(t\right)=t+\frac{1}{\mu_{max}}ln\left[e^{-\mu_{max}t}+e^{{-h}_{0}}-e^{-\mu_{max}t{-h}_{0}}\right]$$

(3) $$y\left(t\right)=y_{0}+y_{max}-ln\left\{e^{y_{0}}+\left[e^{y_{max}}-e^{y_{0}}\right]e^{-\mu_{max}B\left(t\right)}\right\}$$

(4) $$B\left(t\right)=t+\frac{1}{4}ln\frac{1+e^{-4\left(t-\lambda \right)}}{1+e^{4\lambda }}$$

(5) $$y\left(t\right)=y_{0}+\left(y_{max}{-y}_{0}\right)exp\left\{-exp\left[\frac{\mu_{max}e}{y_{max}{-y}_{0}}\left(\lambda -t\right)+1\right]\right\}$$

where *y*(*t*) represents the bacterial concentration at time *t*, expressed as the natural logarithm of colony-forming units per gram (ln CFU/g); *y*_0_ is the initial bacterial concentration (ln CFU/g) at the start of the growth phase; *yₘₐₓ* is the maximum bacterial concentration (ln CFU/g) reached during the stationary phase; *μₘₐₓ* denotes the maximum specific growth rate (h^−1^); *t* is the time (h); *λ* refers to the duration of the lag phase (h); and *h*_0_ describes the initial physiological state of the bacteria in response to environmental conditions.

#### 2.6.2. Secondary Models

To evaluate the effect of temperature (*T*) on the kinetic parameters, *μ_max_*, λ, and *y_max_* obtained from the primary model at different incubation temperatures were fitted to the secondary model. The effect of temperature on *μ_max_* was evaluated using the Ratkowski square root (Equation (6)) [33] and Huang square root models (Equation (7)) [34].

(6) $$\sqrt{\mu_{max}}=a\left({T-T}_{0}\right)\left[1-e^{b\left({T-T}_{max}\right)}\right]$$

(7) $$\sqrt{\mu_{max}}{=a\left({T-T}_{min}\right)}^{0.75}$$

where *μ_max_* is the maximum specific growth rate of bacterial growth (h^−1^); *T* is the environmental temperature the bacteria are exposed to (°C); *T*_0_ is the minimum growth temperature of bacteria (°C); *T_max_* is the maximum growth temperature of bacteria (°C); a and b are coefficients.

#### 2.6.3. Probabilistic Model

The probability generated by BA is fitted using the Logistic model; the results of BA concentration ≧ 0.25 mg/kg were assigned as 1, otherwise 0, and the formula is as follows:

(8) $$logit\left(P\right)=ln\frac{P\left(x\right)}{1-P\left(x\right)}=\sum b_{i}x_{i}$$

In the equation, *P* refers to the probability of BA producing/not producing (range 0–1), *x_i_* is the influencing factor, and *b_i_* is the parameter value to be estimated.

### 2.7. Model Validation

Internal and external validation were performed to evaluate the model developed to predict isothermal growth of BGC on FWRN. For the internal validation, the predicted data were compared with the observed data. For the external validation, we compared the predicted data with the observed data from our independent experiments. All validations were performed using the correlation coefficient (R^2^), accuracy factor (*A_f_*), bias factor (*B_f_*), and root mean square error (RMSE), calculated according to Equations (9)–(12), respectively.

(9) $$R^{2}=1-\frac{\sum_{i=1}^{n}{\left(pred-obs\right)}^{2}}{\sum_{i=1}^{n}{\left(obs-mean\right)}^{2}}$$

(10) $$A_{f}=10^{\left[\frac{\sum_{i=1}^{n}\left|log\left(pred/obs\right)\right|}{n}\right]}$$

(11) $$B_{f}=10^{\left[\frac{\sum_{i=1}^{n}log\left(pred/obs\right)}{n}\right]}$$

(12) $$RMSE=\sqrt{\frac{\sum_{i=1}^{n}{\left(y_{i}-{ŷ}_{i}\right)}^{2}}{n}}$$

where *y_i_* and *ŷ_i_* are the observed and predicted microbial populations (log CFU/g) or specific growth rate (h^−1^), n is the number of observations, obs is the observed value, and pred is the predicted value.

The predictive models developed in this study were validated using both internal and external datasets. The internal validation involved comparing the model predictions with the experimental data used for model fitting under the various temperature conditions.

For external validation, an independent batch of fresh wet rice noodle (FWRN) samples (*n* = 15) was collected from a separate production lot obtained from a different local manufacturer to represent natural variability in raw material and processing conditions. These FWRN samples were subjected to the same BGC inoculation and storage protocols as the primary experimental set, covering three representative temperatures (10 °C, 24 °C, and 30 °C) that were not included in the internal modeling dataset. Each temperature condition included five replicates to ensure statistical robustness (5 samples per temperature × 3 temperatures = 15 samples total).

The external validation data were compared against the model-predicted bacterial counts using statistical indicators including root mean square error (RMSE), bias factor (*B_f_*), and accuracy factor (*A_f_*). Additionally, the coefficient of variation (CV) and adjusted coefficient of determination (R^2^adj) were calculated to further assess the model’s predictive performance while accounting for variability within the external dataset. The inclusion of these additional parameters provided a more comprehensive evaluation of model fit and predictive capability.

This external validation approach, utilizing independent samples with varied origins and contamination levels, aimed to strengthen the model’s applicability to real-world conditions and ensure its robustness across different FWRN production and storage scenarios.

The validation of the predictive models in this study was performed through both internal and external validation procedures. The internal validation relied on cross-comparison with experimental data obtained under controlled laboratory conditions, while the external validation was conducted using independent experiments based on the same batch of FWRN samples. While these methods confirmed the accuracy and reliability of the models under the tested conditions, we acknowledge that the external validation did not yet incorporate samples from diverse geographical origins or varying contamination levels. Given the potential variability in raw material quality, microbial background, and processing conditions across different production sites, future studies will include a wider variety of FWRN samples collected from multiple regions and manufacturers. Additionally, we will introduce varying initial contamination levels and simulate different environmental stresses during storage and transportation to further evaluate the model’s robustness and adaptability. Expanding the scope of validation will enhance the reliability and applicability of the predictive models in real-world scenarios and strengthen their role in food safety risk assessment for FWRN.

## 3. Results and Discussion

### 3.1. Growth Kinetic Model of BGC in Fresh Wet Rice Noodle and Validation

**Figure 2:**
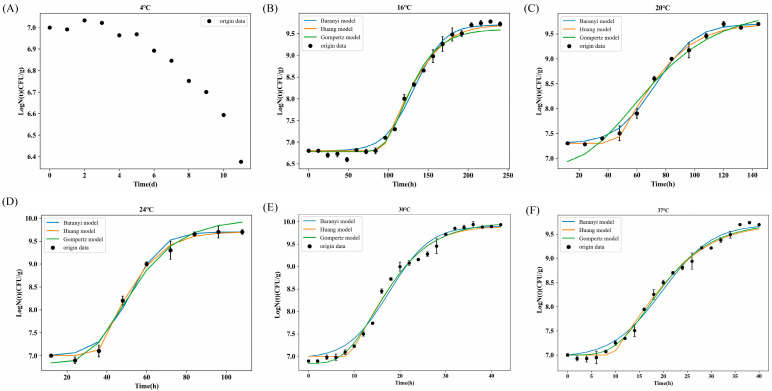
The experimental concentration of BGC in FWRN and growth curves fitted with the Baranyi, Huang, and modified Gompertz models. (**A**) 4 °C; (**B**) 16 °C; (**C**) 20 °C; (**D**) 24 °C; (**E**) 30 °C; (**F**) 37 °C.

Figure 2 displays the concentration of BGC in FWRN stored at varying temperatures and the growth curve that the models have been fitted to. The initial inoculation level was approximately 6.5–7 log CFU/g, and the maximum concentration of BGC after culture reached 9.7–9.95 log CFU/g. At 4 °C, the concentration of bacteria exhibited a declining pattern, presumably due to the bacteria transitioning into the viable but not culturable (VBNC) phase [35], with the data not fitting the parameters of these specific growth models. Hawa Ahmad [36] conducted a one-step kinetic analysis to examine the growth of thermophilic *Bacillus cereus* in liquid egg yolks across a temperature range of 9–50 °C and found similar growth patterns at specific temperatures. No growth of Bacillus cereus was observed in liquid egg yolk, and the number of inoculated bacteria gradually decreased at both 9 °C and 50 °C. Under various types of stress, such as low temperatures and starvation, more than 60 pathogens were reported to be in the VBNC state [37]. In this state, the pathogen remains active but is unable to grow and reproduce under standard ordinary culture conditions. This may lead to difficulties in detecting bacterial contamination and excessive production of toxins by the pathogen, thereby increasing the potential threat of VBNC bacteria to food safety and public health. Therefore, further studies are required to develop novel detection methods that can determine whether BGC has transitioned to the VBNC state at low temperatures. Moreover, it is imperative to implement measures to prevent and manage the development of VBNC bacteria.

The growth curves at 16 °C, 20 °C, 24 °C, 30 °C, and 37 °C exhibited distinct phases of lag, exponential growth, and stabilization. The growth curves of BGC in FWRN during storage were represented by Baranyi, Huang, and modified Gompertz models, respectively, and are illustrated in Figure 2. The optimal growth temperature of BGC in FWRN, ranging from 4 to 37 °C, showed slight variations compared to the prior research. Additionally, in this study, the growth rate of BGC in FWRN at varying temperatures was assessed through microbial prediction modeling. The results revealed that the highest growth rate (*μ_max_*) of 0.2603 log CFU/g/h occurred at 30 °C, suggesting that 30 °C is the most favorable growth temperature for BGC. However, Li et al. [38] showed that the optimal growth temperature for BGC was 37 °C, but that study was based on absorbance values rather than actual colony count data. In general, the optimal growth temperature of foodborne pathogens is typically similar to the body temperature of their most common host, the human body. However, *Burkholderia gladiolus* thrives better at lower temperatures, specifically at 30 °C. The reason for this may be that these bacteria originate from diverse environments, including bodies of water, soil, or other organisms, and have adapted to thrive and reproduce at comparatively low temperatures.

In this study, predictive microbiology modeling was employed to investigate the growth of BGC in FWRN at temperatures ranging from 4 °C to 37 °C. Three primary models were utilized to fit the growth rates. Parametrically, the maximum specific growth rate (*μ_max_*) and lag period duration (λ) of the growth curves at different temperatures were evaluated. (Table 1). Using the data presented in the table, the model fitting revealed that the *μ_max_* of BGC falls within the range of 0.0159–0.2603 log CFU/h. At temperatures other than the optimal growth temperature of 30 °C, the growth rate *μ_max_* of BGC gradually increased, while λ significantly decreased with increasing storage temperature. Specifically, the model estimated that the λ of BGC at 16 °C was 95.43–111.01 h, whereas the λ at 37 °C was 9.46–13.82 h, indicating a nearly tenfold difference. There are many factors affecting the length of the λ, including environmental variables and strain differences [39]. After analyzing the impact of environment on production kinetic parameters, it has been found that temperature significantly affects λ, as evidenced by its rapid reduction with an increase in temperature [40]. *Bacillus cereus* is a foodborne pathogen frequently detected in rice and noodle products. Hwang and Huang [25] utilized a one-step kinetic analysis to establish a three-stage model that accurately characterized the growth and survival of Bacillus cereus in cooked rice. The results revealed that the optimal growth temperature was 37.6 °C, and the best specific growth rate was 2.21 ln CFU/g/h or 0.96 log CFU/g/h. Various models exhibit distinct fitting behaviors. For instance, the Huang model exhibits an acute angle at the transition point between the lagging and exponential phases, and it strives to clearly demarcate these two phases [34]. In Nurul Hawa Ahmad’s study, the growth curves of thermophilic Bacillus cereus between 15 and 45 °C did not exhibit a distinct lag phase, and the bacteria initiated exponential growth immediately upon inoculation into liquid egg yolks [36]. This suggests that the Huang model without λ is appropriate for representing these growth curves. Although the BGC exhibited a notable shift in the range of 16–37 °C, the growth data were effectively fitted by all three primary models presented graphically.

RMSE values that are close to 0 suggest that the model predictions are in close proximity to the experimental data, thereby indicating a better model fit. Typically, an acceptable range for RMSE is considered to be between 0.05 and 0.15 [41]. In this study, Huang’s and Baranyi’s models exhibited RMSE values ranging from 0.07 to 0.15, with both models boasting R^2^ values exceeding 0.98. In contrast, the modified Gompertz models exhibited R^2^ values exceeding 0.99, but their RMSE values fell between 0.3 and 0.48. As a result, when juxtaposed with the modified Gompertz model, the experimentally constructed Huang and Baranyi models proved more adept at fitting the BGC growth and offered more precise predictions. In recent years, *Staphylococcus aureus* and *Bacillus cereus* have also been frequently detected in a variety of rice and flour products. Huang et al. [42] and Juneja et al. [43] examined the development of Staphylococcus aureus and Bacillus cereus in glutinous rice dough and cooked rice, respectively. Both studies demonstrated that the Gompertz model outperformed the Huang and Baranyi models at extreme temperatures ranging from 13–19 °C to 40–46 °C. However, it is worth noting that all models exhibited satisfactory performance at optimal growth temperatures. In our study, there was minimal disparity in the performance of the three models at 16 °C. To ensure the reliability and reproducibility of the experimental data, all bacterial growth experiments under each temperature condition were independently repeated three times. The growth parameters presented in Table 1, including μmax and lag phase duration (λ), are expressed as mean values of the triplicate measurements. The variability within the replicates was accounted for by calculating the standard deviation (SD) for each parameter. The corresponding SD values are provided alongside the mean values in Table 1 to present the data variability transparently. These statistical measures improve the interpretation of experimental uncertainty and allow for a more accurate assessment of model fitting across different temperature conditions.

**Figure 3:**
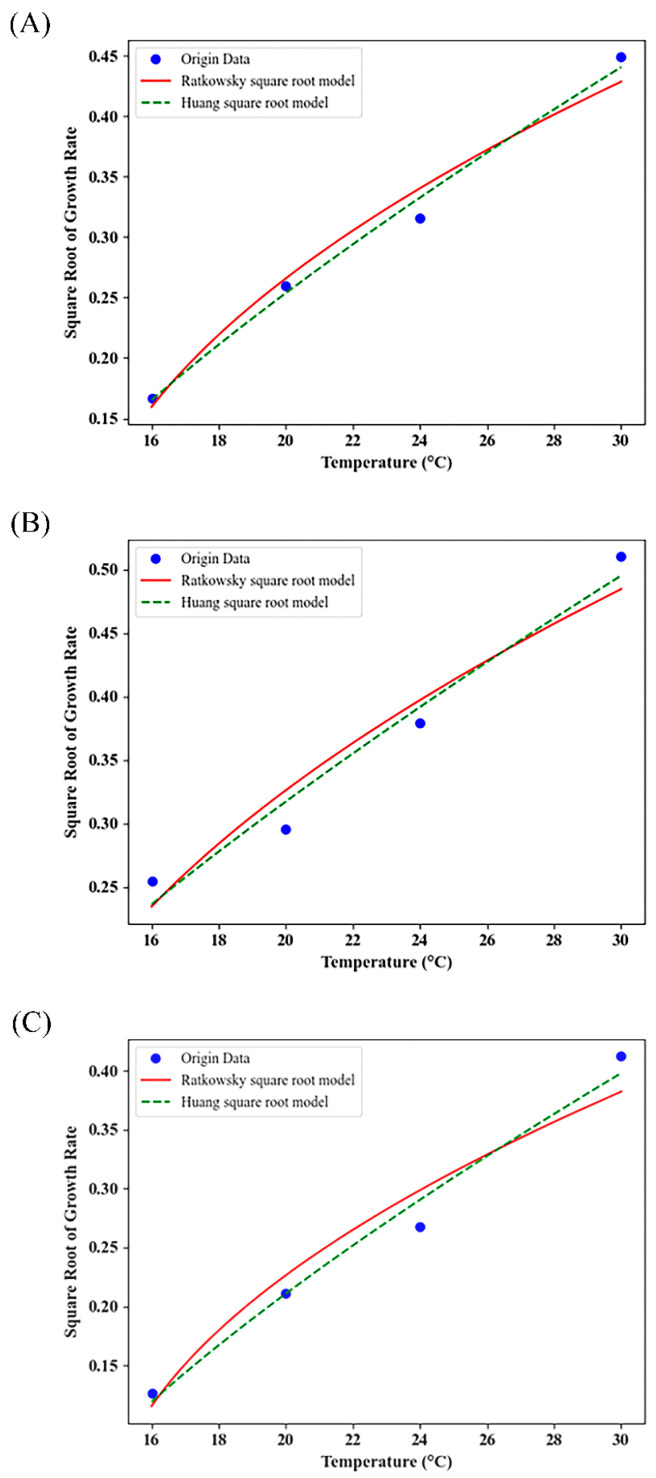
Effect of temperature on the maximum growth rate (*μ_max_*) of BGC in FWRN. (**A**) based on Huang model; (**B**) based on Baranyi model; (**C**) based on modified Gompertz model.

The Ratkowsky square root model and Huang square root model were used in the study to evaluate the effect of temperature on the *μ_max_* of BGC, and the graphs (Figure 3) visualized that the relationship between the temperature and the root *μ_max_* is showing a partial linear relationship. Table 2 presents the evaluation of the prediction models for the specific growth rate of BGC in FWRN. When *A_f_* and *B_f_* are within the range of 0.9–1.05, the accuracy factor of the model is high and the error is minimal. Between 0.70–0.90 and 1.06–1.15, the model’s accuracy was deemed acceptable, yet its error was substantial. If 0.70 or greater than 1.15, the model is deemed unreliable and cannot be employed for simulations that aim to depict conditions like microbial growth. As can be observed from Table 2, with the exception of the modified Gompertz combined with the Ratkowsky square root model, the *A_f_* and *B_f_* values for the remaining model combinations were much closer to 1. The estimated minimum growth temperatures (*T_min_*) using the Huang square root model ranged from 7.39 to 12.51, while those calculated using the Ratkowsky square root model spanned from 11.58 to 14.59. It is important to recognize that the nominal minimum growth temperatures (*T*_0_) are the estimated theoretical minimum growth temperatures, which are typically lower than the minimum growth temperature in the actual environment. Furthermore, the experiments have confirmed that the BGC can thrive at 10 °C. As a result, the Huang square root model was ultimately selected as the secondary model for the growth of BGC in FWRN to explain the impact of temperature on *μ_max_*. For the study of the growth of *Staphylococcus aureus* in roasted oysters, Ma et al. [44] constructed model combinations of the Huang–HSR, Baranyi–HSR, and two-compartment–HSR. These model combinations exhibited similar fitting effects, but the Huang model proved to be simpler than the Baranyi and two compartment models. Therefore, the Huang–HSR model combination is the recommended choice. In conclusion, the Huang model combined with the Huang square root was ultimately selected in this study to characterize the growth of BGCs in FWRN.

The selection of the Huang primary model, coupled with the Huang square root secondary model, aligns with the findings of Lu et al. [45], who systematically evaluated multiple modeling approaches for predicting the growth of Staphylococcus aureus in ready-to-eat meat loaf rice balls. Their comparative analysis revealed that the Huang model combination outperformed alternative models in terms of goodness-of-fit and predictive reliability, suggesting its suitability for describing bacterial growth kinetics under complex food matrices and varying environmental conditions.

In this study, the Huang model was ultimately selected as the primary model for predicting the growth kinetics of BGC in FWRN, primarily due to its lower RMSE values and consistently high R^2^, *A_f_*, and *B_f_* values across different temperature conditions. Although the Baranyi and modified Gompertz models also demonstrated acceptable fits, the Huang model showed superior performance in delineating the transition between the lag phase and exponential phase, which is particularly relevant to BGC’s growth characteristics observed in this study. However, we recognize that additional model selection criteria, such as the Akaike Information Criterion (AIC) and Bayesian Information Criterion (BIC), would provide a more comprehensive evaluation by balancing model complexity against the goodness-of-fit. Future research will incorporate AIC, BIC, and potentially other statistical indicators to strengthen the model selection process and further validate the robustness of the Huang model under varied conditions. This approach will enhance the objectivity and reproducibility of model choice in predictive microbiology applications.

Although this study noted the potential for *Burkholderia gladioli* to enter a viable but nonculturable (VBNC) state at low temperatures (e.g., 4 °C), this state was not directly confirmed due to the lack of specific detection methodologies within the experimental design. The absence of VBNC-targeted detection could result in an underestimation of the actual viable cell population, as conventional plate counts cannot capture cells that are metabolically active but nonculturable. This limitation could affect the accuracy of the growth kinetic models, especially under low-temperature conditions, and may underestimate the potential food safety risks associated with VBNC cells, which retain the capacity to resuscitate or produce toxins under favorable conditions. To address this limitation, future work will integrate molecular techniques such as quantitative PCR (qPCR), reverse transcription qPCR (RT-qPCR), or propidium monoazide-qPCR (PMA-qPCR), which can distinguish between viable and dead cells and detect VBNC bacteria with greater sensitivity. Applying these methods will provide a more comprehensive understanding of BGC survival dynamics and its associated risks in FWRN, particularly under cold storage scenarios where VBNC induction is likely.

### 3.2. Quality Changes in Fresh Wet Rice Noodle During Storage

**Figure 4:**
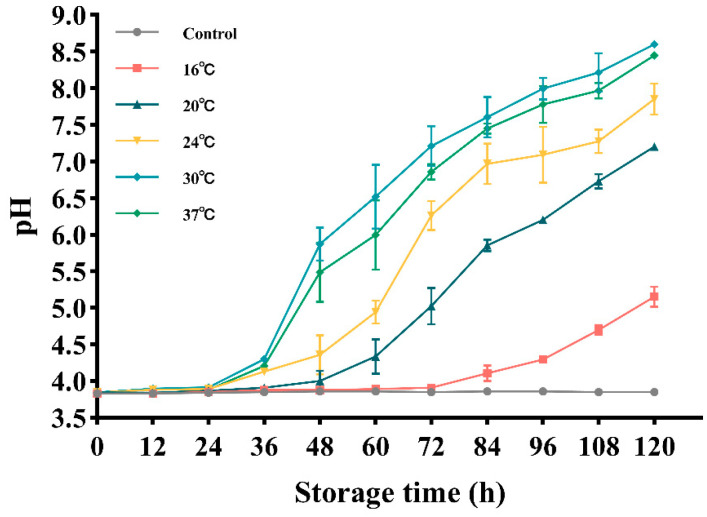
The pH changes in FWRN when stored at 16 °C, 20 °C, 24 °C, 30 °C, and 37 °C after being contaminated by BGC.

Due to the action of BGC, the moisture content, hardness, pH, and color values of FWRN will undergo changes during storage, and the rate and extent of these changes will have an impact on the quality of FWRN. Hence, the quality of FWRN contaminated by BGC was assessed, and the parameters of moisture content, hardness, pH value, and color value were determined. The results revealed that the moisture content and hardness of FWRN remained largely unchanged, displaying no notable differences, standing at 64.5% and 46.7 N, respectively. The initial pH of FWRN employed in the experiments was approximately 3.8. As depicted in Figure 4, over time, the pH value of the blank control group of FWRN remained unchanged. However, the pH of FWRN contaminated by BGC exhibited an increasing trend at 16 °C, 20 °C, 24 °C, 30 °C, and 37 °C. Furthermore, except for 30 °C, the higher the temperature, the higher the pH value of BGC-contaminated FWRN for a consistent storage duration. Due to contamination by a single colony during storage of FWRN, relatively few studies have been conducted on the changes in quality at different temperatures. The primary focus of the current research is on the long-term storage of FWRN and the impact of temperature on its overall quality. Observations from researchers Yang et al. [46] and Qiao et al. [47] indicate a decreasing trend in moisture content, brightness, and acceptability of FWRN when stored at 25 °C. However, the variations in hardness and pH were slightly dissimilar. The degree of regrowth of FWRN is directly reflected by hardness, and alterations in FWRN hardness are primarily influenced by the growth and reproduction of microorganisms as well as starch regrowth. The reduction in water content in FWRN may be associated with the consumption of water by microbial metabolism, along with the transfer of water within FWRN and the freezing of free water. In their study, Yang et al. [46] discovered that the initial pH of FWRN was approximately 4.98 at the start of the storage period. Over the subsequent two weeks, the pH value rose to approximately 5.10. Interestingly, this change was independent of the storage temperature, and the pH value remained stable throughout the storage period. This result highlights the stabilizing effect of an acidic environment on the long-term storage of FWRN [48]. In contrast, Qiao et al. [47] discovered that the hardness of FBRN significantly increased at various storage temperatures, whereas the pH and moisture content decreased as storage time increased. Microbial activity is often responsible for altering the pH levels of carbohydrate-rich foods. This occurs when microorganisms consume carbohydrates and produce acids, resulting in a decrease in pH. Furthermore, according to Li et al. [49], as pH decreases, fresh noodles undergo protein decomposition, which releases alkaline compounds such as amines and ammonia. This, in turn, leads to an increase in pH.

Color is a crucial indicator of the quality of FWRN. The L*, a*, and b* values were measured using a colorimeter, where the L* value represents brightness, ranging from 0 to 100 (from black to white). The value of a* indicates the transition from red to green, which corresponds to a range of −120 to +120. The value of b* denotes the transition from yellow to blue, where positive values correspond to yellow and negative values correspond to blue. The patterns of changes in L*, a*, and b* values of BGC-contaminated FWRN over storage time at various storage temperatures are illustrated in Table 3 and Table 4. The table reveals that storage time and temperature significantly impact the appearance and color of FWRN. The range of L* values for all treatment groups remained consistently between 70 and 73. The b* values of FWRN in the blank control group and at 16 °C remained almost constant throughout the storage period, with no significant difference observed between them. During the pre-storage period at 20 °C, 24 °C, 30 °C, and 37 °C, the variations in b* values of FWRN within each group were minimal. However, as the storage time increased, the b* values of FWRN progressively rose, leading to a noticeable divergence among the groups. Additionally, FWRN gradually turned yellow as the storage time extended. The maximum color change was observed at 30 °C, and the yellowness of FWRN increased with higher storage temperatures, except at 30 °C. Our findings are in line with those of Xue et al. [28] and Yang et al. [50]. The browning of fresh wet rice flour may be associated with the enzymatic activities of peroxidase and polyphenol oxidase. Increased temperatures lead to an uptick in enzymatic activity, resulting in a decrease in brightness and a rise in browning over time during storage. The observed color changes in BGC-contaminated FWRN, particularly the gradual increase in b* values (yellowing) and fluctuations in a* values (red–green axis), have practical significance for consumer perception and acceptance. In the context of fresh rice noodles, consumers typically associate whiteness (higher L* values) and minimal color deviation with freshness and high quality. The increase in b* values, reflecting a yellowing tendency over time, is likely to be perceived as a sign of product deterioration or spoilage, which may negatively affect consumer purchase intent and sensory satisfaction.

While this study focused on instrumental colorimetric data, sensory perception involves more complex and subjective visual assessments. Therefore, future work will incorporate sensory analysis panels to evaluate consumer perception of FWRN appearance under different contamination and storage scenarios. Such sensory trials will help to establish threshold values for color changes (e.g., Δb* or ΔL*) that correspond to noticeable visual deterioration from a consumer perspective. The integration of objective color measurements with sensory evaluation will offer a more comprehensive understanding of how microbial spoilage and quality degradation influence marketability and consumer acceptance of FWRN.

The Pearson correlation coefficient, which is a more widely used linear correlation coefficient, can indicate the extent of the correlation between two random variables. Table 5 displays the results obtained from the evaluation of the correlation between the CFU level of BGC in fresh wet rice flour at various storage temperatures and times and each quality index, utilizing Pearson’s correlation coefficient. The significance of *p* < 0.01 for the CFU level with temperature, pH, and b* values suggests a correlation between CFU and temperature, pH, and b. The correlation coefficients for CFU with temperature, pH, and b* values are r = 0.774, r = 0.699, and r = 0.627, respectively, all of which are close to 1. This suggests that the level of CFU is positively correlated with temperature, pH, and b* values.

The Pearson correlation analysis between BGC CFU levels and FWRN quality parameters (temperature, pH, L*, a*, and b*) provides preliminary insights into the relationships among these variables. While significant positive correlations were observed between CFU levels and factors such as temperature (r = 0.774) and pH (r = 0.699), correlation alone does not imply causality. The multifactorial nature of food spoilage and microbial growth suggests that these quality attributes may be influenced by complex interactions involving microbial activity, temperature, and time. Therefore, the conclusions derived from the correlation matrix should be interpreted with caution.

To further strengthen the inference of causality and better quantify the contributions of individual variables to microbial proliferation and quality changes, a multivariate regression analysis will be conducted in future work. This approach will enable us to control for potential confounding variables and evaluate the independent effects of factors such as pH and color parameters on BGC growth dynamics. Additionally, integrating advanced statistical methods, such as principal component analysis (PCA) or partial least squares regression (PLSR), could provide deeper insights into the multivariate relationships and further improve the robustness of the predictive models.

In this study, the quality assessment of FWRN primarily focused on instrumental parameters, including pH, color (L*, a*, b*), texture (hardness), and moisture content, which are widely recognized as key indicators of rice noodle quality. However, we acknowledge that a more comprehensive analysis could be achieved by including additional microbiological and sensory parameters. In real-world settings, the spoilage and safety of FWRN are not only affected by BGC but may also involve interactions with other microorganisms commonly present in rice-based products, such as Bacillus cereus, Staphylococcus aureus, and lactic acid bacteria.

To enhance the practical relevance of this study, future research will expand the microbiological evaluation to include total viable counts (TVC), lactic acid bacteria counts, and the presence of common foodborne pathogens. This will provide a more holistic understanding of microbial ecology in FWRN under different storage conditions. Furthermore, sensory analysis—including assessments of appearance, odor, texture, and overall acceptability—will be incorporated to bridge the gap between instrumental measurements and consumer perception. Such enhancements will enable the development of a more complete risk and quality assessment framework, ensuring that both microbial safety and sensory quality are fully addressed in FWRN shelf-life studies.

### 3.3. Production and Probabilistic Model of Bongkreic Acid

**Figure 5:**
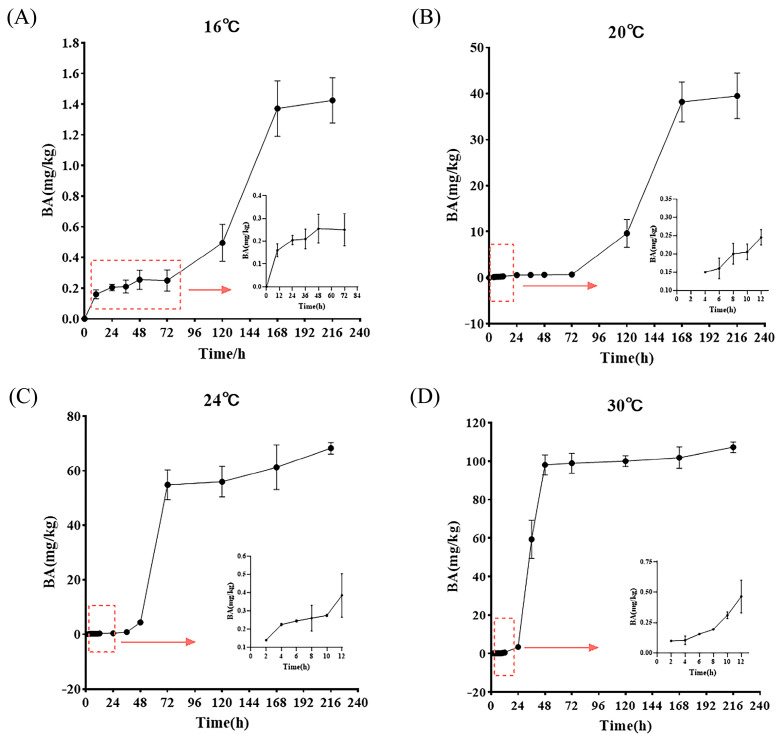
BA accumulation in FWRN at different storage temperatures. (**A**) 16 °C; (**B**) 20 °C; (**C**) 24 °C; (**D**) 30 °C.

Figure 5 illustrates the production of BA by BGC at varying storage temperatures over a 2 h to 9 d incubation timeframe. At lower temperatures (e.g., 4 °C), BA production was limited, leading to undetectable levels of BA, which are not shown in Figure 5. Meanwhile, as illustrated in Figure 2, BGC growth stagnates at 4 °C. During the storage period, the production of BA gradually increased and eventually stabilized over time, a trend that remained unaffected by temperature. Furthermore, there was a notable disparity in the ultimate concentration of BA as it stabilized at varying temperatures in contrast to BGC growth. Initially, we only measured the amount of BA produced by the BGC on days 1, 2, 3, 5, 7, and 9, and all of these measurements exceeded the limit of 0.25 mg/kg. Thus, by optimizing the timing of detection, we were able to supplement the data with BA produced by BGC during the initial 24 h. At 16 °C, BA was detected as early as 10 h, with its production ranging from 0.16 to 1.43 mg/kg during the incubation time. The detected BA content at 10 h and 24 h was 0.16 mg/kg and 0.21 mg/kg, respectively, and thereafter exceeded the limit value of 0.25 mg/kg. Furthermore, Figure 2 showed that when FWRN was contaminated with BGC and stored at 16 °C for 24 h, the BGC was in the delayed phase, at which time the bacterial concentration was 10^7^ CFU/g. This finding again emphasized that bacterial concentration and toxin production are not linearly related. The optimal temperature range for BA production was between 24 °C and 30 °C. A significant and sudden increase in BA production was observed at 72 h at 24 °C and 36 h at 30 °C, with rates of increase reaching 12.5-fold and 18-fold, respectively. Under these temperature conditions, the highest accumulation of BA was observed at 30 °C, reaching 107.17 mg/kg. With the exception of 4 °C and 16 °C, the accumulation at varying temperatures had surpassed the limit value of 0.25 mg/kg around 10 h. This suggests that when the limit value of BA is low and the concentration of BGC is high, a greater amount of BA production takes place in a brief time frame.

**Figure 6:**
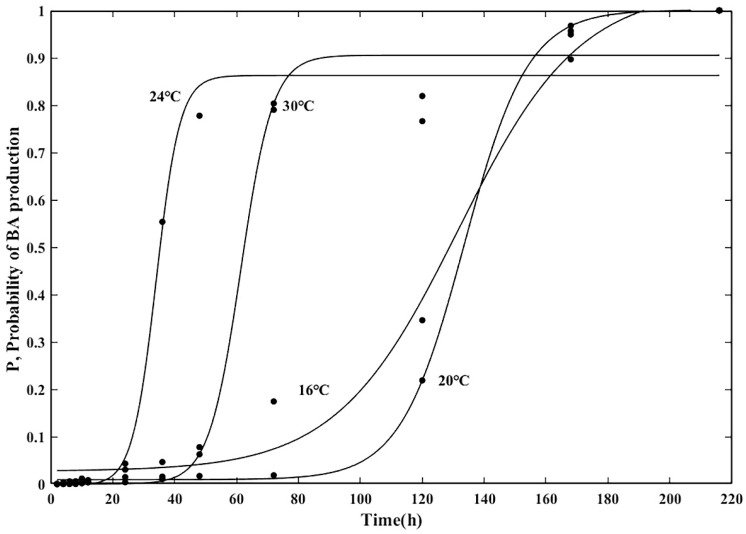
Probabilistic model of BA production in FWRN.

**Figure 7:**
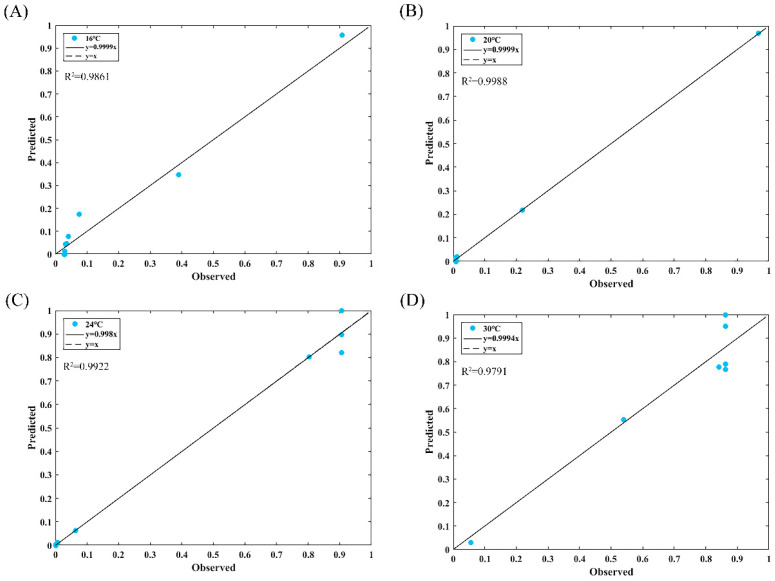
Logistic model predicted value-observed value correlation plot. (**A**) 16 °C; (**B**) 20 °C; (**C**) 24 °C; (**D**) 30 °C.

Based on Figure 5, it is observed that there is a significant increase in BA production over time, and ultimately, BA accumulation stabilizes. Hence, it is not feasible to explain the production of BA over time using a generalized linear model. To illustrate the impact of temperature on BA production, the data were analyzed and simulated using a logistic model in MATLAB 2020a, with the condition that BA concentration results ≧ 0.25 mg/kg at different incubation temperatures were assigned as 1 and 0 otherwise. The impact of temperature on BA production was elucidated by converting the results of miscanthus acid concentration into production probability. Figure 6 displays the predicted probability of BA production over time under various conditions. At constant temperatures, the probability of growth gradually increased over time, indicating that the strain exhibits greater potential for growth under these conditions. Meanwhile, Figure 7 displays the correlation plots of the model’s predicted and observed values, revealing that the R^2^ values for these predicted–observed values remain above 0.97 across various temperatures, thereby underscoring the model’s superior fit. While the probabilistic model developed in this study effectively captured the production dynamics of BA within the tested temperature range (16 °C to 30 °C), it is important to acknowledge potential limitations in extrapolating the model to extreme or untested conditions, such as sub-refrigeration temperatures (≤4 °C) or elevated ambient temperatures (>37 °C). Predictive models based on limited environmental conditions may not fully account for non-linear biological responses or stress adaptations that BGC may exhibit under such extremes, leading to potential under- or over-estimation of BA production risks.

To address these limitations, future work will incorporate simulated scenarios extending beyond the experimental range. Specifically, predictive simulations at temperatures such as 2 °C, 10 °C, and 40 °C will be performed using Monte Carlo methods or extended logistic modeling. These simulations will allow for stress testing the robustness and reliability of the model under broader environmental conditions. Additionally, experimental validation under such extreme scenarios will be integrated to refine and recalibrate model parameters where necessary, ensuring greater predictive accuracy across a wider spectrum of storage and transportation conditions.

Up to now, there has been a relatively limited amount of research conducted on the environmental factors that contribute to the production of BA from BGC and its predictive status. Furthermore, BGC is frequently identified in uncooked rice and flour. Rice and wheat are often contaminated with fungi and molds, in addition to bacteria, resulting in the production of secondary metabolites. As a result, the results were analyzed in comparison to *Aspergillus* and *Fusarium flavus*, along with their production of secondary metabolites in wheat and rice. Upon comparing the BA production probability model with the BGC growth model, it was discovered that the temperature range for toxin production is narrower than the temperature range for strain growth. This suggests a disparity between microbial growth and toxin production. Although colony growth may occur in certain instances, it does not necessarily entail toxin production, and this observation also applies to fungi and their mycotoxins. Rather, the production of toxin may be influenced by environmental factors and stress conditions. The results of this study are in line with the findings of other researchers. In the present study, we did not detect BA at 4 °C, whereas BGC stagnated at this temperature. Garcia-Cela et al. [51] discovered that temperature and water activity (10–35 °C; aw, 0.87–0.98) have an impact on the growth and toxin production of three *Fusarium* species in wheat, including Zearalenone, Deoxynivalenol, and Nivalenol. They discovered that the range of environmental conditions that supported mycotoxin production was narrower than that of conditions supporting colony growth. For instance, Fusarium exhibited visible growth at 10 °C, whereas Nivalenol and Zearalenone were not detected at 20 °C, 15 °C, or 10 °C. However, it has also been discovered that the strain can produce a wider range of toxins than it can grow, allowing for adaptation to lower moisture levels. The production of mycotoxins is often advocated as an adaptive response to suboptimal growth conditions [52]. For instance, in chestnuts, while drying at 40 °C notably reduced fungal growth, it could also enhance aflatoxin production [53,54]. In addition, Norlia et al. [55] used peanut meal extract agar to study the effects of temperature and moisture activity on *A. flavus* growth and aflatoxin production, and model predictions yielded optimal temperatures for the highest growth rates of *A. flavus* strains in the range of 32–33 °C and optimal temperatures for aflatoxin production in the range of 25–30 °C, which were in agreement with the optimal temperatures for growth reported by previous researchers on a variety of substrates, such as rice [56] and pistachio [52]. The result is comparable to the optimal growth and toxin production temperature ranges of BGC in fresh wet rice flour, with both optimal toxin production temperatures being slightly below the optimal growth temperature.

The external validation results are presented in Table 6, which summarizes the performance of the Huang–Huang combination model across the three independent temperature conditions (10 °C, 24 °C, and 30 °C). In addition to RMSE, *B_f_*, and *A_f_*, two additional metrics were introduced to enhance the evaluation of model performance: the adjusted coefficient of determination (R^2^adj) and the coefficient of variation (CV).

The Huang–Huang model demonstrated satisfactory predictive capability across all external conditions, with R^2^adj values ranging from 0.964 to 0.978, indicating a strong model fit while adjusting for the degrees of freedom associated with each dataset. The CV values, ranging from 6.2% to 8.5%, suggest acceptable levels of variability between observed and predicted values across different validation scenarios. These findings further corroborate the reliability and robustness of the model when applied to independent datasets outside the original modeling range.

The inclusion of R^2^adj and CV provides a more comprehensive assessment of model accuracy and consistency, supporting the application of the Huang–Huang model for BGC growth prediction in FWRN under diverse storage conditions.

## 4. Conclusions

The objective of this study was to examine the ecological behavior of BGC in FWRN, employing various primary and secondary models to elucidate the impact of temperature on the growth of BGC. The performance of the primary models was evaluated using R^2^ and RMSE, and it was discovered that the Huang and Baranyi models exhibited a superior fit for the BGC growth in comparison to the modified Gompertz model. The Huang square root model, which is based on the Huang model, particularly exhibited superior performance with an RMSE value of 0.009 and *A_f_* and *B_f_* values of 1.062 and 0.998, respectively.

During growth experiments, it was discovered that BGC exhibited the fastest growth rate within the 30–37 °C range, indicating that this temperature range is the most optimal for BGC growth. Furthermore, experiments on toxicity production indicated that the optimal temperature for BA production is 26–30 °C. Therefore, it is recommended to strictly control the temperature of both processing and storage environments, particularly avoiding storage and processing in the temperature range that is most suitable for the growth of BGC. Simultaneously, the storage and usage period of FWRN is designed to minimize its storage time. Although low-temperature storage (4 °C) prevents the growth of BGC and toxin production, it may also cause changes in the taste and texture of FWRN, affecting its flavor characteristics. For instance, it may result in hardening upon water absorption and increased strip breakage. Furthermore, when FWRN are newly contaminated with BGC or when the concentration of contamination is low, no discernible signs of deterioration may be apparent. Hence, any alteration in the color of FWRN, particularly the emergence of a yellow hue, should be regarded as a reason for alarm. Once any abnormalities are detected, it is advisable to immediately discard the unsuitable fresh wet rice flour to prevent any potential food safety risks.

Although this study recommends avoiding storage and transportation of FWRN within the temperature range of 24 °C to 30 °C due to the elevated risk of BGC proliferation and BA production, we recognize that maintaining such control may be challenging in real-world supply chains, particularly in regions with limited cold-chain infrastructure or during warm seasons. To improve the practical applicability of our recommendations, economically and technically feasible strategies should be considered.

For instance, implementing cost-effective cold-chain interventions, such as maintaining storage temperatures at or below 10 °C using standard refrigeration units or insulated containers during short-term transportation, can effectively reduce BGC growth rates. Additionally, the adoption of modified atmosphere packaging (MAP) to limit oxygen availability, combined with low-temperature storage, may further inhibit microbial proliferation and toxin production. The use of time–temperature indicators (TTIs) on packaging can also help monitor and manage temperature deviations during distribution. By integrating such preventive strategies into the supply chain, producers and distributors can better mitigate the risks identified in this study without incurring excessive operational costs, thereby enhancing food safety while maintaining product quality.

## Figures and Tables

**Table 1:** Growth parameters of growth of BGC in fresh wet rice noodles evaluated with different primary models (mean ± SD).

Model	*T* (°C)	*μ_max_* (log CFU/g/h) ± SD	λ (h) ± SD	RMSE	R^2^
Huang	16	0.0280 ± 0.0021	95.43 ± 4.8	0.0755	0.9965
	20	0.0676 ± 0.0035	45.46 ± 3.2	0.0716	0.9945
	24	0.1003 ± 0.0047	34.66 ± 2.6	0.0574	0.9975
	30	0.2023 ± 0.0061	8.93 ± 0.7	0.1075	0.9916
	37	0.1633 ± 0.0053	9.46 ± 0.8	0.0755	0.9965
Baranyi	16	0.0655 ± 0.0038	111.01 ± 5.5	0.1214	0.9909
	20	0.0818 ± 0.0045	58.66 ± 3.9	0.0839	0.9924
	24	0.1442 ± 0.0063	42.47 ± 2.7	0.1276	0.9877
	30	0.2603 ± 0.0072	12.13 ± 1.1	0.1437	0.985
	37	0.2206 ± 0.0068	13.82 ± 1.3	0.1073	0.9893
Modified Gompertz	16	0.0159 ± 0.0017	97.21 ± 6.0	0.4888	0.9929
	20	0.0444 ± 0.0025	23.18 ± 1.8	0.5763	0.9704
	24	0.0717 ± 0.0039	30.73 ± 2.1	0.406	0.9862
	30	0.1698 ± 0.0054	7.69 ± 0.6	0.4846	0.9923
	37	0.1404 ± 0.0047	9.73 ± 0.9	0.3665	0.9941

*μ_max_* (log CFU/g/h): specific growth rate (h^−1^); *T*: temperature (°C); λ: lag phase duration (h). RMSE: root mean square error (ln CFU/g); R^2^: correlation coefficient (ln CFU/g).

**Table 2:** Evaluations of models for predicting the specific growth rate of BGC in fresh wet rice noodles.

Primary Model	Secondary Model	Parameters			Evaluation		
		a	*T_min_*	*T*_0_	RMSE	*A_f_*	*B_f_*
Huang	Huang	0.002	10.81	-	0.009	1.062	0.998
	Ratkowsky	0.011	-	13.74	0.016	1.114	1.002
Baranyi	Huang	0.002	7.39	-	0.014	1.102	0.998
	Ratkowsky	0.012	-	11.58	0.021	1.148	1.004
Modified Gompertz	Huang	0.002	12.51	-	0.014	1.109	0.992
	Ratkowsky	0.009	-	14.59	0.023	1.223	1.223

a: coefficient; *T_min_*: estimated minimum temperature (°C); *T*_0_: nominal minimum temperature (°C). RMSE: root mean square error (h^−1^); *B_f_*: bias factor; *A_f_*: accuracy factor. The dash (-) indicates data not measured or not applicable.

**Table 3:** Color changes in FWRN contaminated with BGC when stored at 16 °C and 20 °C.

Storage Time (h)	Control			16 °C			20 °C		
	L*	a*	b*	L*	a*	b*	L*	a*	b*
0	71.90 a	−2.90 a	−2.21 a	71.90 a	−2.90 ab	−2.21 a	71.90 a	−2.90 a	−2.21 a
12	72.86 a	−2.85 a	−2.57 a	72.24 a	−2.95 ab	−2.24 a	72.24 a	−2.92 a	−1.75 a
24	72.16 a	−2.87 a	−2.31 a	47.32 a	−2.84 ab	−2.04 a	72.31 a	−2.72 a	−1.45 a
36	71.89 a	−2.77 a	−2.65 a	74.28 a	−2.98 a	−2.23 a	71.63 a	−3.12 a	−1.07 a
48	72.06 a	−2.82 a	−2.51 a	72.58 a	−2.90 ab	−1.91 a	71.48 a	−2.33 a	0.16 b
60	71.99 a	−2.76 a	−2.18 a	71.54 a	−2.86 b	−2.14 a	72.08 a	−2.70 a	1.11 bc
72	71.64 a	−2.80 a	−1.95 a	72.07 a	−2.95 ab	−1.60 a	73.08 a	−2.66 a	1.74 c
84	71.74 a	−2.82 a	−2.22 a	73.11 a	−2.94 ab	−2.00 a	73.56 a	−2.85 a	2.58 c
96	71.65 a	−2.84 a	−2.33 a	72.67 a	−2.88 b	−2.17 a	73.22 a	−2.73 a	3.74 c
108	71.82 a	−2.79 a	−2.52 a	72.45 a	−2.95 ab	−2.22 a	72.95 a	−2.68 a	3.89 c
120	71.87 a	−2.88 a	−2.47 a	72.88 a	−2.90 ab	−2.15 a	72.57 a	−2.71 a	4.11 c

Different lowercase letters in the same column indicate significant differences (*p* ≤ 0.05).

**Table 4:** Color changes in FWRN contaminated with BGC when stored at 24 °C, 30 °C, and 37 °C.

Storage Time (h)	24 °C			30 °C			37 °C		
	L*	a*	b*	L*	a*	b*	L*	a*	b*
0	71.90 a	−2.90 cd	−2.21 a	71.73 a	−2.87 b	−2.21 a	71.90 a	−2.90 a	−2.21 ab
12	72.24 a	−2.92 cd	−1.75 ab	71.96 a	−2.92 a	−1.72 a	72.24 a	−2.95 b	−1.74 a
24	72.31 a	−2.72 d	−1.45 ab	70.38 a	−2.95 a	−1.25 b	70.97 a	−2.90 a	−1.32 b
36	71.63 a	−3.12 c	−1.07 ab	72.31 a	−2.93 a	0.76 a	72.15 a	−2.96 a	−1.37 b
48	72.19 a	−3.02 cd	−0.85 b	70.38 a	−3.06 a	3.56 c	70.55 a	−3.26 a	2.39 c
60	74.27 a	−3.95 b	2.89 c	70.55 a	−3.25 a	4.06 c	69.82 a	−3.37 a	2.73 c
72	70.50 a	−4.31 a	3.72 c	71.38 a	−3.31 a	7.20 d	72.21 a	−3.07 a	6.99 d
84	71.42 a	−4.19 a	4.5 cd	70.95 a	−3.29 a	8.90 d	71.28 a	−3.35 a	7.14 d
96	72.31 a	−4.22 a	5.55 d	72.31 a	−3.06 a	10.58 e	69.26 a	−3.16 a	7.87 d
108	71.63 a	−4.17 a	6.25 e	70.38 a	−3.25 a	10.93 e	70.53 a	−3.15 a	8.55 d
120	72.19 a	−4.25 a	6.85 e	70.55 a	−3.31 a	13.0 f	66.24 b	−3.20 a	11.29 e

Different lowercase letters in the same column indicate significant differences (*p* ≤ 0.05).

**Table 5:** Correlation analysis between CFU levels and other factors.

		CFU Levels	Temperatures	Time	pH	L*	a*	b*
CFU levels	r	1						
	*p*							
Temperatures	r	0.774 **	1					
	*p*	0.000						
Time	r	0.287	0.008	1				
	*p*	0.069	0.960					
pH	r	0.699 **	0.518 **	0.696 **	1			
	*p*	0.000	0.001	0.000				
L*	r	0.004	0.024	0.161	0.022	1		
	*p*	0.980	0.884	0.314	0.891			
a*	r	−0.153	−0.171	−0.459 **	−0.620 **	−0.076	1	
	*p*	0.340	0.285	0.003	0.000	0.635		
b*	r	0.627 **	0.408 **	0.624 **	0.922 **	0.043	−0.672 **	1
	*p*	0.000	0.008	0.000	0.000	0.791	0.000	

** At the 0.01 level (two-tailed), the correlation is significant.

**Table 6:** External Validation Of The Huang–Huang Model.

Temperature (°C)	RMSE	*B_f_*	*A_f_*	R^2^adj	CV (%)
10	0.011	1.003	1.045	0.964	8.5
24	0.008	0.997	1.038	0.972	7
30	0.01	1.005	1.042	0.978	6.2

## Data Availability

The authors confirm that the data supporting the findings of this study are available within the article, and the raw data that support the findings are available from the corresponding author, upon reasonable request.

## Abbreviations

The following abbreviations are used in this manuscript:

FWRN	Fresh wet rice noodles
BGC	*Burkholderia gladioli pathovar cocovenenans*
BA	Bongkrekic acid
VBNC	Viable but not culturable
mPDA	Modified potato dextrose agar
*μ_max_*	Maximum specific growth rate
λ	Lag period duration
RMSE	Root mean square error
*A_f_*	Accuracy factor
*B_f_*	Bias factor
